# Knowledge Representation and Management in the Age of Long Covid and Large Language Models: a 2022-2023 Survey

**DOI:** 10.1055/s-0044-1800747

**Published:** 2025-04-08

**Authors:** Jonathan P. Bona

**Affiliations:** Department of Biomedical Informatics, University of Arkansas for Medical Sciences

**Keywords:** Knowledge Representation and Management, Biomedical Ontologies, Artificial Intelligence, Post-Acute Covid-19 Syndrome, Natural Language Processing

## Abstract

**Objectives** : To select, present, and summarize cutting edge work in the field of Knowledge Representation and Management (KRM) published in 2022 and 2023.

**Methods** : A comprehensive set of KRM-relevant articles published in 2022 and 2023 was retrieved by querying PubMed. Topic modeling with Latent Dirichlet Allocation was used to further refine this query and suggest areas of focus. Selected articles were chosen based on a review of their title and abstract.

**Results** : An initial set of 8,706 publications were retrieved from PubMed. From these, fifteen papers were ultimately selected matching one of two main themes: KRM for long COVID, and KRM approaches used in combination with generative large language models.

**Conclusions** : This survey shows the ongoing development and versatility of KRM approaches, both to improve our understanding of a global health crisis and to augment and evaluate cutting edge technologies from other areas of artificial intelligence.

## 1. Introduction

The goal of this survey is to present and summarize cutting edge work in the field of Knowledge Representation and Management (KRM) published in 2022 and 2023. Efforts to understand and combat the ongoing COVID-19 pandemic remain prominent topics in medical informatics. The 2021 Yearbook's Knowledge Representation and Management survey paper highlighted the role of clinical knowledge in the COVID-19 pandemic in moving towards a global learning health system [ 1 ]. The 2022 survey paper focused on KRM work seeking to address health inequities, including those exposed and exacerbated by the COVID-19 pandemic [ 2 ].

Now in the fourth year of the pandemic, KRM approaches continue to be adapted and applied to address many areas of this multifaceted crisis. One example is the Coronavirus Infectious Disease Ontology (CIDO) effort [ 3 ], which provides a semantically rich framework for representing and managing information about coronavirus diseases. The application of KRM approaches to COVID-19 issues includes efforts aimed at the growing health crisis posed by long COVID. One special area of focus for this survey is emerging knowledge representation work that seeks to address the growing health crisis posed by long COVID (“post-COVID syndrome”, “Postacute sequelae of SARS-CoV-2 infection (PASC)”). Characterized by the long-term persistence of symptoms following SARS-CoV-2 infection [ 4 ], and occurring following at least 10% of COVID-19 cases, long COVID is complex, associated with over 200 distinct symptoms [ 5 ] and serious complications [ 6 ]. A January 2023 estimate placed the number of persons afflicted with long COVID worldwide at over 65 million. Long COVID negatively impacts health and quality of life for those afflicted by it [ 7 ] and is creating significant economic burdens and stresses on healthcare systems [ 8 , 9 ]. In the face of this complex and baffling health crisis, there is a critical need for research to collect, organize, integrate, and interpret large and diverse sets of relevant data [ 10 11 12 ]. Knowledge representation approaches are well-poised to help address this need, and this review examines several such efforts now under way.

Another area of special focus in this survey is the growing field of combined use of knowledge representation techniques with generative AI and Large Language Models.

The last two years have seen a revolutionary development in Artificial Intelligence (AI) and Natural Language Processing (NLP): the emergence of generative Large Language Models (LLMs) [ 13 ] with the ability to use and produce natural language at a level of competence far exceeding any previous efforts. OpenAI's Generative Pre-trained Transformer (GPT) [ 14 ] (and its public-facing chat interface ChatGPT) have attracted significant public interest, as well as interest in its capabilities from experts in fields that have already been leveraging AI technologies, including in healthcare and medicine [ 15 16 17 ]. LLMs, and GPT/ChatGPT in particular, have received attention for their potential to assist in medical diagnosis [ 18 , 19 ]. To become such competent users of language, GPT and similar generative LLMs were trained on massive amounts of text harvested from the Internet. This has allowed for the creation of open domain conversational agents like ChatGPT, with an impressive ability to discuss nearly any topic in natural language. Sometimes dismissed as “stochastic parrots” [ 20 ] or, at the other extreme, considered as a harbinger of Artificial General Intelligence (AGI) [ 21 ], these models do show some ability to engage in tasks requiring reasoning. However, their limited ability to understand [ 22 ], propensity to “hallucinate” (make things up) [ 23 ], and limited ability to reason [ 24 ] pose challenges that may best be addressed by solutions that combine LLMs with other AI techniques best-suited for such tasks.

These challenges (understanding, veracity, and reasoning) are all areas that are well suited to solutions from the areas of KRM. The use of these approaches in combination with LLMs is a rapidly emerging field, and this survey highlights recent projects in this area.

## 2. Methods

A comprehensive set of KRM-relevant articles published in 2022 and 2023 was retrieved by querying PubMed with a large disjunctive query including terms “ontology”, “knowledge representation”, “semantic web” and “description logic”, filtered to results from 2022 and 2023 (see query Q1 in Table 1 ). The resulting corpus of 8,706 titles and abstracts was judged too large to be evaluated manually.

**Table 1.** PubMed queries and result counts

Query		#Results
Q1	(„knowledge representation“ OR „description logic“ OR „semantic web“ OR „ontology“ OR „ontologies“)	8,706
Q2	Q1 AND NOT („gene ontology“) NOT „review“[sb] NOT „systematic review“[sb]	1,264

**Figure 1.**
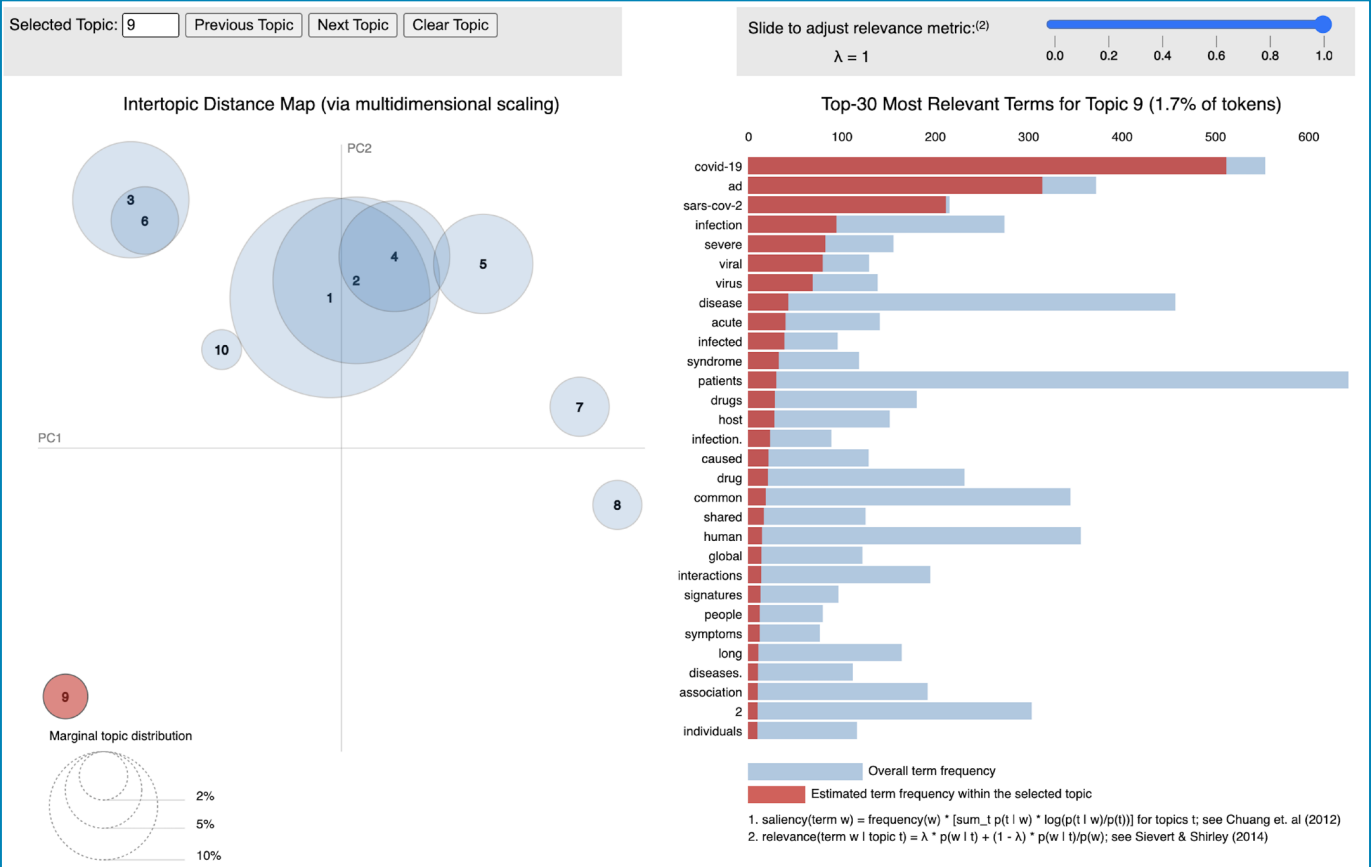
PyLDAVis topic model visualization focused on topic #9 (COVID-19), for which the 30 most relevant terms are highlighted at the right. Numbered circles on the left show the distance between topics when reduced to a 2D space. The marginal topic distribution shows how relatively popular a topic is within the entire corpus.

The corpus was analyzed using topic modeling (Latent Dirichlet Allocation (LDA) [ 25 ], implemented in Python using scikit-learn) to identify significant topics common across large subsets of these abstracts. LDA does not label or categorize the topics it identifies, but presents each topic as a distribution over a set of words ( Figure 1 ). Inspecting the top words for a topic is one way of coming to understand what the topic is about. This topic analysis of the 8700 or so abstracts retrieved by our query identified a particularly relevant topic for this survey, which may best be characterized as “biomedical KRM” (topic #3). Table 2 shows the ten most important terms for this topic, along with two others: discovered by this analysis identifiable as “covid-19” (topic #9) and “cancer” (topic #2), among others (genetic epidemiology (topic #6), genomics (topic #1), non-coding RNA research (topic #7), pharmacology and drug discovery (topic #4)).

**Table 2.** A sample of recognizable abstract topics and their top terms.

Topic#	2	3	9
Description	Cancer	Biomedical KRM	Covid-19
Top 10 Terms	cancer	data	covid-19
	immune	knowledge	ad
	expression	information	sars-cov-2
	genes	health	infection
	analysis	research	severe
	patients	semantic	viral
	prognostic	ontologies	virus
	cell	model	disease
	gene	use	acute
	tumor	approach	infected

A manual inspection of a subset of abstracts within these topics revealed that the widespread use of the Gene Ontology (GO) to annotate scientific literature was a contributing factor to the large amount of search results over this timespan. A second query (Q2) was developed to exclude most mentions of the GO, as well as excluding systematic reviews. The resulting set of abstracts was manually reviewed to identify abstracts matching two main topics of interest: COVID-19, and specifically long COVID, and generative NLP / LLMs.

## 3. Results

### 3.1. Long COVID

Long COVID negatively impacts health and quality of life for the tens of millions of people worldwide who are or have been afflicted by it [ 7 ] and is creating significant economic burdens and stresses on healthcare systems [ 8 , 9 ]. There is a critical need for research to collect, organize, integrate, and interpret large and diverse sets of relevant data [ 10 11 12 ].ledge representation and knowledge management approaches are well-poised to help address this need. Below we highlight a few such reports seeking to clarify our understanding of long COVID through the application of KRM techniques. There is a rich body of KRM work targeting COVID-19 more generally, some of which has been reported in past years' surveys, so we emphasize here recent additions to this literature that are focused on long COVID. Identifiable sub-themes in this section include:

General knowledge representation and knowledge management approaches to handling the massive amount of complex data relevant to developing an understanding of long COVID;

Development and use of ontologies/terminologies to capture, organize, and understand information about the diverse clinical presentations of long COVID;

Use of the GO specifically within analyses that investigate similarities between long COVID and conditions such as myalgic encephalomyelitis/chronic fatigue syndrome (ME/CFS).

Providing an overview of health informatics approaches to managing long COVID, Ambalavanan et al. [ 26 ] highlight the large-scale collection of electronic health record data for the purpose of understanding the COVID-19 pandemic and managing long COVID. Among these efforts in the United States is the National Covid Cohort Collaborative (N3C) [ 27 ] data enclave, which has assembled de-identified medical records including 3 billion observations for over 22 million from across 83 sites [ 28 ]. Ambalavanan et al. review long COVID identification and characterization techniques, endorsing an approach to interpreting health data in pursuit of an understanding of long COVID that includes knowledge representation techniques in combination with other AI techniques in the areas of machine learning and deep learning. Useful for standardizing and managing complex data and knowledge and data are phenotypic ontologies, like the HPO which provides “a hierarchical classification of standardized human pathological features that are used to define phenotypes” [ 29 ].

In an ontology-driven effort to organize emerging information about clinical manifestations of long COVID, Deer et al. [ 30 ] conduct an analysis of almost sixty long COVID studies, identifying 287 distinct clinical manifestations (“signs, symptoms, and laboratory as well as imaging abnormalities”) of long COVID, and mapping these symptoms to terms in the Human Phenotype Ontology. They also create new terms (HPO term synonyms and plain-language definitions) aimed at making this information more accessible to laypersons, and developing common data elements to support interoperability across long COVID studies.

A project with a similar goal, to understand the symptomology of long COVID as expressed in clinical notes, Wang et al. [ 31 ] report on the development of a comprehensive long COVID symptom lexicon based on their analysis of over 300,000 notes from long COVID patients. The approach uses the MTERMS [ 32 ] NLP tool to identify relevant symptoms mentioned in these notes, augmented by chart review and other evaluation. The result is PASCLex, a publicly-available lexicon of 355 long COVID symptom terms, as well as detailed information about the frequency with which these symptoms are mentioned in notes for long COVID patients.

In a long COVID related project with a small KRM component, Lee et al. [ 33 ] seek to identify distinguishing characteristics in the immunological profiles of long COVID and severe COVID patients. This project uses the GO in gene set enrichment ultimately finding differences in DNA methylation between those who developed long COVID and those who did not.

Lv et al. [ 34 ] also conduct GO and pathway enrichment analysis, aimed at identifying common genes among long COVID patients and patients with myalgic encephalomyelitis/chronic fatigue syndrome (ME/CFS). Symptoms experienced by sufferers of ME/CFS and long COVID overlap significantly, and there are a number of similarities in the underlying biology [ 35 ]. This study conducted GO analysis of genes associated with long COVID and ME/CFS, and identified nine common genes, also predicting/proposing candidate drugs that might be used as interventions to treat long COVID based on their interactions with these genes. In a systematic review and analysis seeking to understand the commonalities between long COVID and ME/CFS, Tziastuodi et al. [ 36 ] also examine the genetics of COVID-19 and ME/CFS, combining findings from 71 COVID-19 studies and 26 ME/CFS studies, conducting GO analysis, finding evidence of gene overlap, and suggesting possible interventions to be explored.

In another long COVID project that uses electronic medical record (EMR) data and ontologies, Reese et al. [ 37 ] perform a semantic clustering of nearly 6500 long COVID patients based on multicenter EMR data collected within the National Covid Cohort Collaborative (N3C). N3C data uses the OMOP [ 38 ] common data model. In order to use the HPO with this set of patient data, this project used the OMOP2OBO [ 39 ] algorithm to map N3C OMOP codes to HPO terms. They deployed the Phenomizer algorithm to compute a similarity matrix of long COVID patients based on their phenotypic features expressed in HPO, and organized these patients into clusters using k-means clustering. This analysis yielded six long COVID subtype clusters, which the authors labeled as following based on their characteristic features: multisystem+lab, pulmonary, neuropsychiatric, cardiovascular, pain/fatigue, and multisystem-pain. The approach was developed using a set of data from one of six data partner sites, and then applied to the remaining data to demonstrate generalizability.

### 3.2. Knowledge Representation and Large Language Models

The emergence of generative LLMs [ 13 ] is a major scientific development that has attracted significant public interest. LLMs like GPT/ChatGPT and its competitors are also seeing widespread interest in their use in healthcare and medicine [ 15 16 17 ]. Possessing an unprecedented ability to use and generate natural language text, these tools are nonetheless limited in their ability to perform intelligent tasks involving understanding and reasoning, and are known to “hallucinate” – making up answers rather than trying to figure out what is known to be true. Knowledge representation and knowledge management approaches can help to address these challenges. The use of KRM approaches in combination with LLMs is a rapidly emerging field, and this survey highlights recent projects in this area.

Among projects presented in this survey that combine KRM with LLMs in 2022-2023, a few sub-themes are identifiable, including:

LLM-based natural language question & answer interfaces used to facilitate access to ontology-aligned biomedical datasets;

Use of ontologies as sources of ground truth/knowledge or evaluation of LLM-based solutions;

Use of LLMs to augment GO term enrichment and gene set analyses.

In a topical and recent survey and analysis, Denecke et al. [ 40 ] queried experts in healthcare NLP to study how transformer-based models – LLMs like GPT are the most well-known examples – may shape the practice of healthcare. The contribution of transformer models to healthcare knowledge management was the most immediately relevant among the eight categories of use cases identified in this qualitative analysis. Respondents were also enthusiastic about potential applications in the areas of documentation and clinical coding, and decision support, both areas where integration with existing KRM approaches will likely be fruitful.

The GO is one of the largest and most widely-used biomedical ontologies in the world [ 41 ]. As discussed earlier, GO is so heavily used to annotate genes and gene products in the scientific literature and is also so extensively used for term enrichment in bioinformatics, that explicitly excluding “gene ontology” in literature queries may be necessary to focus those queries on knowledge representation work. As of January 2024, GO has over 40,000 active terms [ 42 ], and in 2023 alone it was referenced in over 4,500 publications indexed by PubMed.

To assist researchers with protein function prediction work using GO, Giri et al. [ 43 ] have developed the GO2Sum tool, which uses an LLM (Text-to-Text Transfer Transformer (T5) [ 44 ]) fine-tuned on GO terms and UniProt descriptions. GO2Sum generates descriptions of protein functions by aggregating and summarizing descriptions of individual genes associated with GO terms. GO2Sum was evaluated by comparing its output to existing functional descriptions in the UniProt database.

Similarly, Hu et al. [ 45 ] seek to facilitate gene set analyses by deploying LLMs as “functional genomics assistants” capable of generating names and summaries for gene sets of interest. This project created a pipeline that uses the standard GPT-4 API, providing an initial prompt that instructs GPT to act as an “efficient and insightful assistant to a molecular biologist”, then given a set of genes and asked to generate biologically descriptive names and analysis text. Unlike the GO2Sum project described above, this use of GPT-4 relies on knowledge latent in the model, and does not use GO terms as inputs. Rather, the GPT pipeline is evaluated by comparing its outputs to knowledge stored in the GO. Note however, that GPT's large training corpus of texts of all sorts harvested from the web, likely includes GO annotations [ 46 ]. The authors of this study conclude that GPT-4 has potential for this use, though in gene set analyses it produces “highly similar names” to the GO 50% of the time, and produces “unverifiable statements” in its analyses 22% of the time. It is worth noting that for projects like this and the preceding, the availability of the GO as a high-quality curated knowledge source appears to be invaluable as a source of truth and comparison.

In a recent PubMed-indexed preprint on related work, with conclusions more critical of LLM-based methods, Joachimiak et al. [ 46 ] report on their development of the Structured Prompt Interpolation of Natural Language Descriptions of Controlled Terms for Ontology Reporting (SPINDOCTOR) method, which performs gene set function summarization using GPT based on different sources of information, including ontologies and latent knowledge baked into the language model. The report concludes that results produced by this LLM-based approach are “typically plausible, relevant, and largely free of hallucination”, but they lack precision and the ability to reliably quantify the relevance of terms and perform worse on less well-known genes. These results highlight the continued need for curated knowledge bases to keep LLM-based projects grounded in reality.

Munarko et al. [ 47 ] report on their development of a tool for using natural language queries to conduct exploratory searches on repositories of data that have been semantically annotated with ontologies, the Biosimulation Model Search Engine (BMSE) [ 47 ]. While they claim generalizability of the approach to other domains for data annnotated using RDF and ontologies, their specific use case is a repository of biosimulation models encoded in CellML and using annotation from ontologies such as the Ontology of Physics for Biology (OPB), the Foundational Model of Anatomy (FMA), and Chemical Entities of Biological Interest (ChEBI). This work builds on the CASBERT mechanism [ 48 ] developed by the same group, which uses the Bidirectional Encoder Representations from Transformers (BERT) [ 49 ] LLM to support text-based searches over ontology-aligned data, as a more accessible alternative to SPARQL queries. Previous work in this area includes SPBERT [ 50 ], which used multilayer bidirectional transformers like BERT for SPARQL query generation and result verbalization, training their models on SPARQL query logs to create a tool that translates natural language queries into SPARQL, and to generate natural language answers based on the query results.

LLMs, and GPT/ChatGPT in particular, have received attention for their potential to assist in medical diagnosis [ 18 , 19 ]. Reese et al. [ 51 ] join this conversation with an ontology-based assessment of GPT-4's performance at differential diagnostic reasoning. The group performed two assessments: one based on de-identified clinical notes, and one based on clinical features extracted from those notes as HPO terms. The second evaluation approach is motivated by noting that, in practice, clinical notes ordinarily cannot be transmitted to ChatGPT because they will contain personally identifiable information. When working with narrative-based text, the performance of GPT-4 at this task was “good” at 38.7%, and similar to the performance reported in Kanjee et al. [ 18 ]. However, on the more practical task of producing diagnoses based on extracted clinical features, GPT performed much worse. The paper concludes with the suggestion to combine use of LLMs with knowledge representation approaches for clinical diagnosis solutions.

Authoring summary notes is a complex and time-consuming process for clinicians. To assist with this, Searle et al. [ 52 ] have developed an approach to generating inpatient Brief Hospital Course (BHC) summaries, pulling together information from a diverse set of source notes written during a patient's hospitalization. The basic approach uses Bidirectional and Auto-Regressive Transformers (BART) [ 53 ]. This is extended by augmenting the model with “guided summarization” by using SNOMED-CT terms for problems and interventions extracted from the notes via more traditional concept extraction approaches. The resulting ontology-guided summarization model is more focused on clinically relevant information and outperforms baseline models at the brief hospital course summarization task with real-world data.

According to Wang et al. [ 54 ], because the use of ontologies and terminologies for rare diseases in clinical data is limited, researchers often need to perform manual phenotyping or apply traditional NLP methods to identify patients with these diseases. To help address this gap, their project fine-tuned LLaMA2 LLMs using training sentences constructed from HPO and OMIM terms. Evaluation of a concept identification task compared standard ChatGPT (3.5) to the fine-tuned LLaMA2 models. ChatGPT performed poorly at this task, hallucinating some terms and concept identifiers that do not exist in the target ontologies.

## 4. Discussion

This survey focuses on 2022 and 2023 papers reporting KRM work in two areas of special relevance currently, namely 1) KRM techniques applied to the emerging long COVID crisis (seven papers), and 2) KRM techniques applied in combination with generative LLMs within the area of medical informatics (eight papers), along with supporting background and other relevant work. The work summarized in this survey shows the impressive versatility of KRM approaches both to improve our understanding of a global health crises and to evaluate and augment cutting edge technologies from other areas of AI.

Work to understand the etiology of long COVID is complicated by the complexity of the disease and its diverse manifestations. The use of ontologies and other KRM approaches in this emerging area provides solutions for organizing, managing, understanding, and using complex information about long COVID, often in combination with complementary approaches from other areas (NLP, semantic clustering, genomics). The recent development and rapid proliferation of generative AI and LLMs are impacting all areas where processing or generation of natural language text is relevant, including widespread interest and applications in many areas of biomedicine. A major limitation in the design of these models is their tendency to confidently produce results without consideration for their accuracy or correctness. Integration of ontologies, knowledge bases, and other KRM approaches with LLMs looks promising as a solution for this problem, providing verifiable curated sources of truth that can improve trust and reliability of systems using these models for biomedical and healthcare applications. LLMs can also help with KRM approaches, by providing a natural interface for humans to interact with complicated tools and resources without requiring deep knowledge of ontologies or related technologies.
